# Adherence to the planetary health diet index and metabolic dysfunction-associated steatotic liver disease: a cross-sectional study

**DOI:** 10.3389/fnut.2025.1534604

**Published:** 2025-02-20

**Authors:** Xin Qiu, Shuang Shen, Nizhen Jiang, Donghong Lu, Yifei Feng, Guodong Yang, Bangde Xiang

**Affiliations:** ^1^Department of Hepatobiliary Surgery, Guangxi Medical University Cancer Hospital, Nanning, China; ^2^Department of Gastroenterology, The First Affiliated Hospital of Guangxi Medical University, Nanning, China; ^3^Guangxi Medical University Cancer Hospital, Nanning, China; ^4^Key Laboratory of Early Prevention and Treatment for Regional High Frequency Tumor, Ministry of Education, Nanning, China; ^5^Guangxi Key Laboratory of Early Prevention and Treatment for Regional High Frequency Tumor, Nanning, China

**Keywords:** dietary pattern, planetary health diet, metabolic dysfunction-associated steatotic liver disease, epidemiology, sustainable diet

## Abstract

**Backgrounds:**

Adherence to the Planetary Health Diet Index (PHDI) has been shown to benefit both individual health and the planet. However, its impact on Metabolic Dysfunction-Associated Steatotic Liver Disease (MASLD) remains unclear. This study aimed to investigate the relationship between PHDI adherence and the MASLD risk.

**Methods:**

We analyzed a cohort of 15,865 adults (aged ≥18 years) using data from the National Health and Nutrition Examination Survey (NHANES, 2005–2018). The PHDI was derived from 24-h dietary assessments and comprised the scores of 15 food groups. Multivariate logistic regression was used to investigate the association between PHDI and MASLD, while restricted cubic spline (RCS) regression and threshold analysis were employed to explore potential non-linear relationship. Subgroup analyses were conducted to assess the influence of various demographic and clinical characteristics on the observed associations. Mediation analysis was performed to evaluate the indirect effect of PHDI on MASLD, and weighted quantile sum (WQS) regression was used to assess the influence of individual PHDI nutrients on MASLD.

**Results:**

Among the cohort, 6,125 individuals were diagnosed with MASLD. Multivariate logistic regression revealed that a higher quintile of PHDI was significantly associated with reduced MASLD risk in the fully adjusted model (OR = 0.610, 95%CI 0.508–0.733, *p* < 0.001). Notably, nonlinear relationships between PHDI and MASLD risk were observed through RCS analysis (*p* = 0.002). Subgroup analyses indicated that PHDI was particularly effective in reducing MASLD risk among females, those with higher education attainment, and those living with a partner. WQS regression identified saturated fatty acids as the most significant factor contributing to MASLD risk (weight = 0.313). Additionally, BMI and waist circumference (81.47 and 87.66%, respectively) partially mediated the association between PHDI and MASLD risk, suggesting that the effect of PHDI on MASLD operates, in part, through its impact on BMI and waist circumference. The association between PHDI and MASLD remained robust across multiple sensitivity analyses.

**Conclusion:**

Our findings indicate that adherence to PHDI is linked to a lower risk of MASLD, providing crucial insights for strategies aimed at mitigating the MASLD epidemic while simultaneously fostering environmental sustainability.

## 1 Introduction

Metabolic dysfunction-associated steatotic liver disease (MASLD), previously known as nonalcoholic fatty liver disease (NAFLD), affects approximately one-third of the global adult population (1). MASLD can progress to metabolic dysfunction-associated steatohepatitis (MASH), advanced fibrosis, cirrhosis, and even hepatocellular carcinoma (HCC), imposing a significant social burden (2). In addition, MASLD is associated with an increased risk of various extrahepatic conditions, including cardiovascular disease, diabetes, and chronic kidney disease (3). The pathophysiology of MASLD is complex, with insulin resistance playing a central role that leads to increased hepatic uptake and accumulation of fatty acids (4). Genetic predispositions and environmental factors often intensify this process (5), driving the progression from simple steatosis to more advanced liver diseases. Therefore, targeted interventions for MASLD have become a pressing necessity.

The global food system today is a primary driver of climate change, generating approximately 26% of all greenhouse gas emissions from human activities, consuming about 70% of the world’s freshwater, and using nearly 40% of available land (6, 7). In the meanwhile, dietary factors rank among the top three risk factors for global diseases in both men and women (8). The interconnected challenges of climate change and diet-related disease burdens underscore the pressing need for a more sustainable food system that provides a nutritious diet meeting essential nutrient requirements. To address these problems, the EAT-Lancet Commission recommended a sustainably produced, calorie-optimized planetary health diet in 2019 (7). This diet emphasizes primarily plant-based foods, limited animal-based products, unsaturated fats in place of saturated fats, and restricts refined grains, processed foods, and added sugars. Adoption of this dietary pattern is believed to help prevent premature deaths and reduce the incidence of diseases, including cardiovascular disease and type 2 diabetes (9). A worldwide transition to this diet could mitigate the dietary impact on global greenhouse gas emissions, land use, and freshwater consumption (10). Based on this planetary health diet, the Planetary Health Diet Index (PHDI) was proposed by Marchioni et al. in 2021 (11). The PDHI consists of the scores of two types of food components: adequacy components (foods to be consumed more) and moderation components (foods to be consumed less). It has been associated with improved overall dietary quality and a lower carbon footprint Adherence to the PHDI was also linked to reduced risk of various diseases, such as metabolic syndrome (12), cardiovascular disease (13), and asthma (14). However, the role of this sustainable dietary index in managing MASLD remains unclear.

Although several dietary patterns, such as the Mediterranean Diet (MD) (15, 16), Plant-based diets (PBDs) (17), and Healthy Eating Index (HEI) (18) have been reported to correlate with MASLD risk, these indices do not simultaneously consider the impact of diet on both human health and the environment. This oversight may result in missed opportunities for addressing the broader implications of dietary choices on global sustainability and public health. In contrast, the PHDI provides a comprehensive, scientifically grounded framework that addresses the challenges of modern dietary patterns while considering both environmental and health concerns. Therefore, exploring the role of the PHDI in the prevention and management of MASLD is of considerable importance.

This study aims to investigate the association between the PHDI and MASLD. By analyzing a large-scale population-based cohort, we seek to clarify the potential role of the PHDI in the prevention and management of MASLD, thereby providing a scientific foundation for clinical practice. Additionally, our findings will contribute to the promotion of sustainable dietary patterns, aligning individual health with environmental sustainability.

## 2 Methods

### 2.1 Survey description

NHANES is conducted biennially to obtain a representative sample of the U.S. civilian population not residing in institutions. Utilizing a multistage probability sampling method, NHANES tracks health and nutritional trends over time. The study received ethical approval from the National Center for Health Statistics (NCHS) Ethics Review Board, and all participants provided informed consent. All procedures complied with applicable guidelines and regulations.

### 2.2 Study population

This study included a cohort of 67,364 NHANES participants from 2005 to 2018. Rigorous exclusion criteria were implemented to enhance result accuracy. Initially, individuals under 18 years old were excluded. Subsequently, participants who were pregnant or accompanied with viral hepatitis, autoimmune hepatitis, and liver cancer were removed. Finally, participants with missing information on alcohol or key variables for calculating PHDI and fatty liver index (FLI) were excluded.

### 2.3 Dietary data

Trained interviewers employed the United States Department of Agriculture (USDA) Automated Multiple Pass Method to collect 24-h dietary recall data (19). Participants were instructed to recount all foods and beverages consumed the previous day, aided by measuring tools to estimate portion sizes. The second dietary recording was conducted by phone, 3–10 days after the initial face-to-face session. Dietary recall data were integrated into the Food Patterns Equivalent Database (FPED), which categorizes foods into the 37 USDA Food Pattern Components using a food composition table (20). Total energy intake was calculated as the average of 2 days of reported dietary intake.

### 2.4 Assessment of MASLD

Steatotic liver disease (SLD) is characterized by the use of FLI, which demonstrates both high sensitivity and specificity (21). The formula is as follows (22):


$$FLI=\frac{e^{\left(0.953\times Ln\left(TG\right)+0.139\times BMI+0.718\times Ln\left(GGT\right)+0.053\times WC-15.745\right)}}{1+e^{\left(0.953\times Ln\left(TG\right)+0.139\times BMI+0.718\times Ln\left(GGT\right)+0.053\times WC-15.745\right)}}*100$$

TG, triglyceride; BMI, body mass index; GGT, gamma-glutamyl transferase; WC, waist circumference.

Participants with an FLI of 60 or higher were categorized as having a high likelihood of SLD (21, 23). Although this diagnostic accuracy is slightly lower than that of biopsy and imaging, it is more suitable for large-scale population screening due to its non-invasive nature and lack of associated trauma. Alcohol intake was evaluated through a 24-h dietary recall (24). Individuals who consumed fewer than 20 grams of alcohol daily (females) or under 30 grams per day (males) were categorized as light drinkers. MASLD was defined as the presence of SLD alongside light alcohol consumption, accompanied by at least one of the cardiometabolic risk factors as previously described (25).

### 2.5 Calculation of PHDI

Compared to the MD, PBDs, and the HEI, the PHDI primarily focuses on promoting both human health and planetary sustainability, with enhanced global applicability and scoring flexibility. The calculation of PHDI was performed through dietaryindex package (26). Thirty-five FPED components are originally reported in volumetric measures (cup-equivalents, ounce-equivalents, and teaspoon-equivalents), and were subsequently converted into grams and computed the average intake over 2 days for each FPED component. Then we chose total energy intake from individual food intake and 15 components (whole grains, starchy vegetables, nonstarchy vegetables, whole fruits (excludes fruit juice), dairy products, red and processed meats, poultry, eggs, fish, nuts and seeds, nonsoy legumes, soy products, unsaturated fatty acids, saturated fatty acids, and added sugar) from the converted FPED components to calculate PHDI in dietaryindex package. Each food group received a score ranging from 0 (minimum) to 10 (maximum) except nonsoy legumes and soy product, which were scored on a 0 to 5 scale, based on daily consumption levels that reflect the greatest health benefits of each food group (13, 27). Scores were assigned proportionally for intakes between the minimum and maximum levels, a method consistent with other dietary indices (28). The possible range of the total score of PHDI is 0 to 140 points.

### 2.6 Covariates

Covariates were identified through a comprehensive review of the literature and clinical expertise. These variables included sociodemographic characteristics such as age, sex, race, education level, family poverty income ratio (PIR), marital status, and smoking history. Clinical indicators, including alanine aminotransferase (ALT), aspartate aminotransferase (AST), and total cholesterol, were also considered. Furthermore, diabetes, hypertension, and cardiovascular outcomes were evaluated. The classifications of race, PIR, education level, and BMI, as well as the definitions of smoking status, diabetes, hypertension, and cardiovascular outcomes, are detailed in Supplementary Data Sheet 1.

### 2.7 Statistical analysis

Continuous variables are reported as mean (standard deviation), while categorical variables are presented as frequency (percentage). PHDI values were stratified into quintiles (Q1-Q5) for further analysis, with the lowest quintile (Q1) serving as the reference. Weighted multivariable logistic regression models were carried out to compute odds ratio (OR) and their corresponding 95% confidence intervals (CI) for measuring the association between the PHDI and MASLD. Three logistic regression models were constructed: Model 1 (unadjusted), Model 2 (adjusted for age, gender, and race), and Model 3 (adjusted for age, gender, race, education, PIR, marital status, smoking, ALT, AST, diabetes, hypertension, cardiovascular outcomes, and total cholesterol). Missing data were addressed using multiple imputation techniques (29). Restricted cubic spline (RCS) analysis was employed to investigate potential nonlinear associations between PHDI and MASLD. A threshold analysis was conducted to identify possible inflection points. Interaction and subgroup analyses assessed whether the relationships were influenced by factors such as age, gender, race, education, marital status, BMI, hypertension, diabetes, and cardiovascular outcomes. A mediation analysis was performed to assess the indirect impact of PHDI on MASLD, with BMI and waist circumference serving as mediators. Weighted quantile sum (WQS) regression was utilized to investigate the effect of individual nutrients within the PHDI on MASLD.

To verify the robustness of our findings, several sensitivity analyses were conducted. First, we examined the association between the PHDI and MASLD for the period from 2011 to 2018 to evaluate its consistency across different timeframes. Next, we tested the PHDI-MASLD association using unweighted data to assess the impact of sample weighting on our results, ensuring that our findings are not biased by weighted estimates. Third, we repeated the analysis with unimputed data, excluding observations with missing variables to explore the association within different population subsets. Finally, we utilized the first day diet data to validate the PHDI-MASLD association, ensuring that the observed relationship is not affected by the averaging method of the two-day data.

All data analyses were performed using R software (version 4.4.0)^1^. Statistical significance was defined by a *p*-value of less than 0.05.

## 3 Results

### 3.1 Baseline characteristics

**Figure 1:**
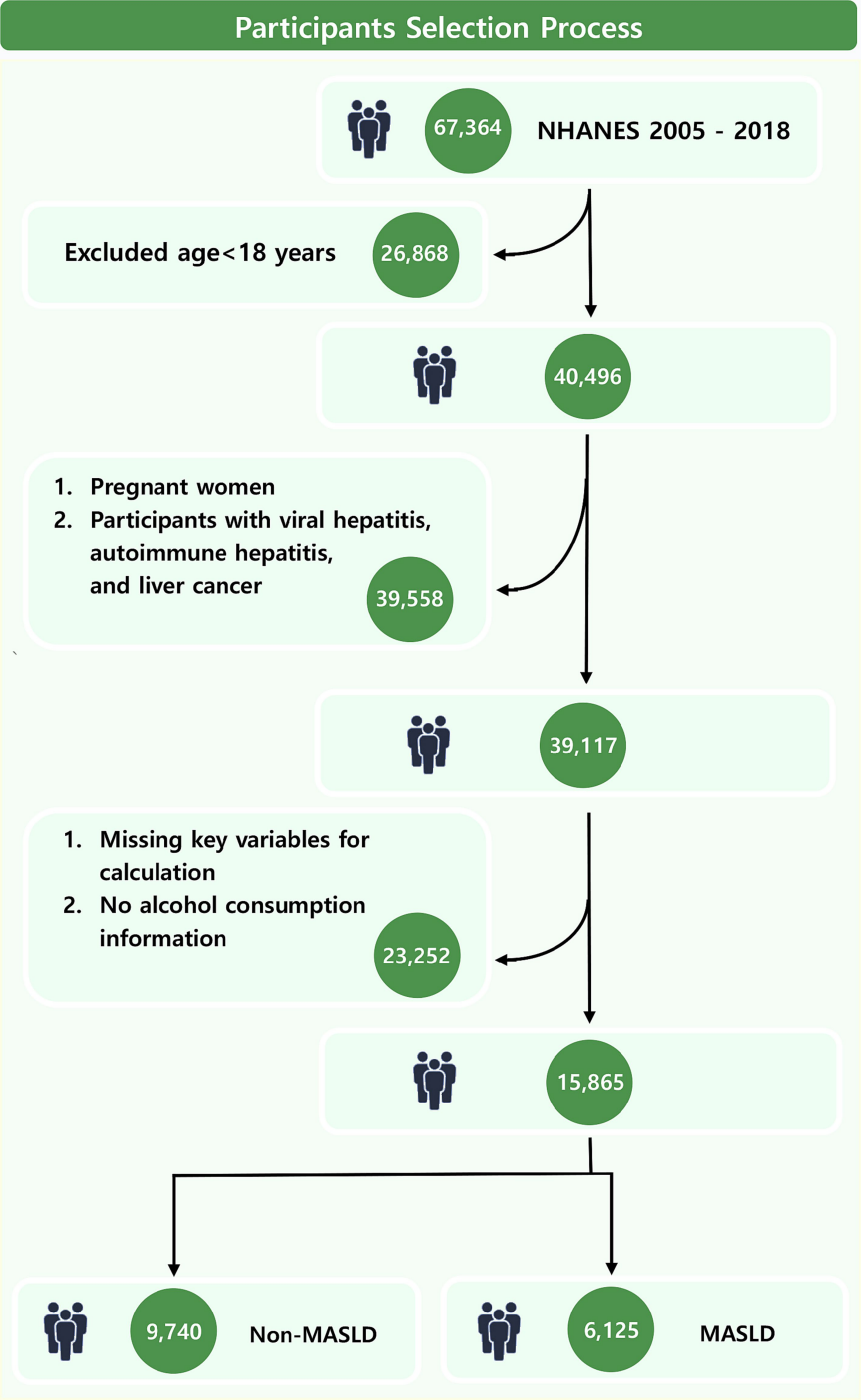
Flowchart of participants selection process.

This study included 15,865 adults (Figure 1) with a median PHDI score of 66.75. Participants in the highest quintile (Q5) of PHDI were predominantly aged 40–59 years, female, married or living with partner, with higher educational attainment and income levels, and a lower prevalence of smoking (all *p* < 0.001) (Table 1). Additionally, individuals in the highest quintile demonstrated significantly lower levels of BMI, lymphocytes count, neutrophils count, and triglycerides (all *p* < 0.001). A total of 6,125 participants (38.61%) were diagnosed with MASLD. In contrast, MASLD patients tended to be male, possess lower educational qualifications, and exhibit higher values of BMI, lymphocytes count, neutrophils count, and triglycerides (all *p* < 0.001) (Supplementary Table S1). Notably, the median PHDI value in MASLD patients was significantly lower than in non-MASLD participants (65.61 vs. 68.09, *p* < 0.001). Furthermore, the prevalence of MASLD was significantly lower among participants in the highest PHDI quintile compared to those in the lowest quintile (Q1) (29.97% vs. 40.93, *p* < 0.001), emphasizing the need for further investigation into the protective effects of PHDI.

**Table 1:** Baseline characteristics of the study.

Characteristics	Overall *N* = 15,865 (100%)^1^	Q1 *N* = 3,173 (20%)^1^	Q2 *N* = 3,173 (20%)^1^	Q3 *N* = 3,173 (19%)^1^	Q4 *N* = 3,173 (20%)^1^	Q5 *N* = 3,173 (21%)^1^	*p* value^2^
Age							<0.001^***^
18–39 years	5,813 (39.22%)	1,538 (52.59%)	1,344 (43.88%)	1,117 (36.79%)	1,003 (33.99%)	811 (29.28%)	
40–59 years	5,011 (35.64%)	941 (31.49%)	956 (37.10%)	1,030 (36.15%)	993 (35.80%)	1,091 (37.50%)	
> = 60 years	5,041 (25.14%)	694 (15.93%)	873 (19.01%)	1,026 (27.06%)	1,177 (30.21%)	1,271 (33.22%)	
Gender							<0.001^***^
Male	7,880 (49.57%)	1,910 (61.95%)	1,691 (52.64%)	1,535 (47.50%)	1,466 (46.63%)	1,278 (39.54%)	
Female	7,985 (50.43%)	1,263 (38.05%)	1,482 (47.36%)	1,638 (52.50%)	1,707 (53.37%)	1,895 (60.46%)	
Race							<0.001^***^
Non-Hispanic White	6,785 (66.65%)	1,287 (64.05%)	1,376 (66.33%)	1,357 (64.57%)	1,403 (68.30%)	1,362 (69.81%)	
Non-Hispanic Black	3,241 (11.00%)	831 (14.68%)	695 (12.53%)	630 (11.33%)	579 (9.24%)	506 (7.39%)	
Mexican American	2,620 (8.95%)	511 (9.48%)	555 (9.49%)	572 (10.20%)	552 (8.85%)	430 (6.85%)	
Other Race	3,219 (13.39%)	544 (11.79%)	547 (11.65%)	614 (13.90%)	639 (13.61%)	875 (15.95%)	
Marital status							<0.001^***^
Married/Living with partner	9,367 (62.27%)	1,726 (55.42%)	1,791 (58.58%)	1,907 (63.78%)	1,930 (65.98%)	2,013 (67.42%)	
Never married	3,244 (19.90%)	864 (27.55%)	765 (23.12%)	588 (17.52%)	543 (15.28%)	484 (16.19%)	
Widowed/Divorced/Separated	3,254 (17.82%)	583 (17.02%)	617 (18.29%)	678 (18.70%)	700 (18.74%)	676 (16.39%)	
Education							<0.001^***^
High school or equivalent	3,597 (23.20%)	824 (26.88%)	832 (27.39%)	719 (25.03%)	716 (22.01%)	506 (14.94%)	
Less than high school	3,935 (16.88%)	963 (23.48%)	869 (19.12%)	800 (17.39%)	726 (13.84%)	577 (10.86%)	
Some college or more	8,333 (59.92%)	1,386 (49.63%)	1,472 (53.49%)	1,654 (57.58%)	1,731 (64.15%)	2,090 (74.20%)	
PIR							<0.001^***^
High income	3,859 (34.11%)	573 (28.34%)	596 (25.87%)	751 (32.34%)	886 (40.43%)	1,053 (43.30%)	
Low income	5,691 (27.02%)	1,394 (33.90%)	1,282 (32.11%)	1,148 (28.34%)	1,020 (21.85%)	847 (19.21%)	
Medium income	6,315 (38.87%)	1,206 (37.76%)	1,295 (42.02%)	1,274 (39.32%)	1,267 (37.72%)	1,273 (37.50%)	
Smoking							<0.001^***^
Now	3,235 (21.22%)	971 (31.14%)	777 (27.22%)	664 (22.24%)	505 (15.86%)	318 (10.09%)	
Former	3,766 (24.21%)	639 (21.41%)	715 (20.90%)	753 (24.45%)	838 (26.30%)	821 (27.88%)	
Never	8,864 (54.57%)	1,563 (47.46%)	1,681 (51.87%)	1,756 (53.30%)	1,830 (57.84%)	2,034 (62.02%)	
Diabetes							0.010^*^
No	13,082 (87.04%)	2,672 (88.93%)	2,636 (87.71%)	2,572 (84.71%)	2,583 (86.03%)	2,619 (87.77%)	
Yes	2,783 (12.96%)	501 (11.07%)	537 (12.29%)	601 (15.29%)	590 (13.97%)	554 (12.23%)	
Hypertension							<0.001^***^
No	11,456 (75.41%)	2,436 (80.10%)	2,366 (77.79%)	2,246 (72.28%)	2,190 (71.40%)	2,218 (75.49%)	
Yes	4,409 (24.59%)	737 (19.90%)	807 (22.21%)	927 (27.72%)	983 (28.60%)	955 (24.51%)	
Cardiovascular outcome							0.004^**^
No	14,583 (93.19%)	2,943 (95%)	2,914 (93.30%)	2,900 (91.95%)	2,911 (93.34%)	2,915 (92.37%)	
Yes	1,282 (6.81%)	230 (5%)	259 (6.70%)	273 (8.05%)	262 (6.66%)	258 (7.63%)	
ALT	21 (16, 28)	21 (16, 30)	21 (16, 29)	21 (16, 28)	20 (16, 28)	20 (16, 26)	0.330
AST	22 (19, 27)	22 (19, 26)	22 (18, 27)	22 (19, 27)	23 (19, 27)	23 (20, 27)	
GGT	19 (14, 29)	21 (15, 31)	20 (14, 31)	19 (14, 29)	19 (14, 29)	17 (13, 24)	
ALP	65 (53, 79)	66 (55, 81)	66 (54, 79)	66 (54, 81)	64 (53, 80)	61 (51, 75)	<0.001^***^
Lymphocytes count	1.90 (1.60, 2.40)	2.00 (1.70, 2.40)	2.00 (1.60, 2.40)	2.00 (1.60, 2.40)	1.90 (1.60, 2.40)	1.80 (1.50, 2.20)	<0.001^***^
Neutrophils count	3.70 (2.90, 4.80)	3.90 (3.00, 5.00)	3.90 (3.00, 5.00)	3.80 (3.00, 4.90)	3.70 (2.90, 4.60)	3.40 (2.70, 4.30)	<0.001^***^
BMI	27.67 (23.90, 32.40)	28.40 (24.20, 33.37)	28.20 (24.53, 32.62)	28.10 (24.30, 32.90)	27.42 (23.71, 32.00)	26.40 (23.00, 30.94)	<0.001^***^
Total Cholesterol	189 (164, 217)	187 (161, 215)	187 (163, 215)	188 (163, 219)	192 (166, 220)	189 (165, 217)	0.027^*^
Triglyceride	1.16 (0.80, 1.72)	1.20 (0.85, 1.78)	1.20 (0.82, 1.76)	1.22 (0.84, 1.82)	1.14 (0.77, 1.64)	1.07 (0.71, 1.56)	<0.001^***^
MASLD							<0.001^***^
No	9,740 (62.13%)	1,876 (59.07%)	1,856 (59.08%)	1,895 (58.68%)	1,938 (63.48%)	2,175 (70.03%)	
Yes	6,125 (37.87%)	1,297 (40.93%)	1,317 (40.92%)	1,278 (41.32%)	1,235 (36.52%)	998 (29.97%)	
PHDI	67.02 (57.70, 77.29)	50.13 (46.29, 53.03)	59.52 (57.52, 61.44)	66.92 (65.15, 68.93)	74.53 (72.38, 77.03)	86.85 (82.82, 92.51)	<0.001^***^

1 *n* (unweighted) (%); Median (IQR).

2 Chi-squared test with Rao & Scott’s second-order correction; Wilcoxon rank-sum test for complex survey samples.

*p < 0.05; **p < 0.01; ***p < 0.001.

### 3.2 Association between PHDI and MASLD

After adjusting for vital variables, including age, gender, race, education, PIR, marital status, smoking, ALT, AST, diabetes, hypertension, cardiovascular outcomes, and total cholesterol in Model 3, a significant inverse association was observed between PHDI and MASLD risk (OR = 0.987, 95% CI 0.983–0.990, *p* < 0.001) (Table 2). Accordingly, the highest quintile of PHDI was associated with a 39% reduction in MASLD risk compared to the lowest quintile (OR_Q5 vs. Q1_ = 0.610, 95% CI 0.508–0.733, *p* < 0.001). In addition, a significant decreasing tendency for MASLD was observed when PHDI quintile increase (*p* < 0.001). We then compared the association between PHDI and other well-known dietary indices, such as the Dietary Approaches to Stop Hypertension Index (DASHI), Alternative Healthy Eating Index (AHEI) (30), and the Alternate Mediterranean Diet (AMED) Score (18), with MASLD risk (Supplementary Table S2). After addressing missing values for the other dietary indices, PHDI showed a similarly significant negative association with MASLD risk, comparable to the other indices. However, adherence to PHDI also promotes planetary sustainability, highlighting the unique advantage of this index.

**Table 2:** The association between PHDI and MASLD.

Variable	Model 1		Model 2		Model 3	
	OR (95% CI)	*p* value	OR (95% CI)	*p* value	OR (95% CI)	*p* value
PHDI	0.987 (0.984, 0.990)	<0.001^***^	0.985 (0.982, 0.988)	<0.001^***^	0.987 (0.983, 0.990)	<0.001^***^
PHDI (Quintile)						
Q1	Ref		Ref		Ref	
Q2	0.999 (0.865, 1.155)	0.994	0.964 (0.832, 1.117)	0.621	0.985 (0.834, 1.163)	0.857
Q3	1.016 (0.870, 1.186)	0.839	0.942 (0.808, 1.098)	0.441	0.903 (0.763, 1.069)	0.233
Q4	0.830 (0.706, 0.976)	0.024^*^	0.757 (0.646, 0.886)	0.001^**^	0.745 (0.637, 0.872)	<0.001^**^
Q5	0.617 (0.527, 0.723)	<0.001^***^	0.557 (0.477, 0.650)	<0.001^***^	0.610 (0.508, 0.733)	<0.001^***^
*P* for trend		<0.001^***^		<0.001^***^		<0.001^***^

*p < 0.05; **p < 0.01; ***p < 0.001.

We further examined the associations between individual PHDI components and MASLD risk (Supplementary Table S3). The analysis revealed the highest quintile of scores for whole grains (OR_Q5 vs. Q1_ = 0.829; 95% CI, 0.692–0.993; *p* = 0.041), non-starchy vegetables (OR_Q5 vs. Q1_ = 0.669; 95% CI, 0.554–0.808; *p* < 0.001), whole fruits (excluding fruit juice) (OR_Q5 vs. Q1_ = 0.755; 95% CI, 0.652–0.875; *p* < 0.001), nuts and seeds (OR_Q5 vs. Q1_ = 0.760; 95% CI, 0.610–0.946; *p* = 0.014), and soy products (OR_Q5 vs. Q1_ = 0.710; 95% CI, 0.534–0.944; *p* = 0.019) were inversely correlated with MASLD risk. Interestingly, the highest quintile of the unsaturated fatty acids score was positively associated with MASLD risk (OR_Q5 vs. Q1_ = 1.638, 95% CI 1.356–1.978, *p* < 0.001). Moreover, the effect of increasing PHDI on reducing MASLD risk was greater than that of nearly every individual PHDI component, highlighting the superiority of this composite index in predicting MASLD.

### 3.3 RCS and threshold analysis of PHDI and MASLD

**Figure 2:**
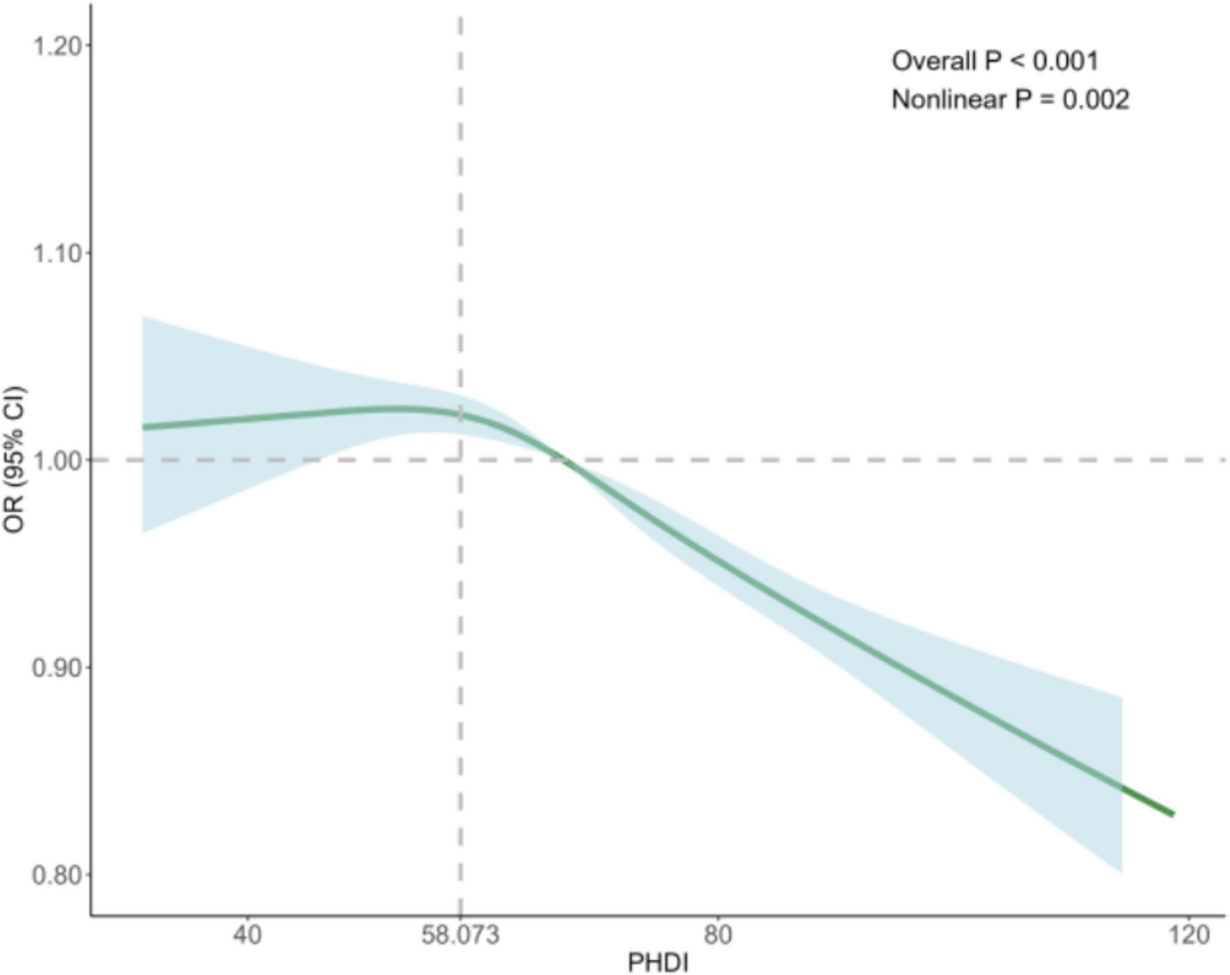
The RCS analysis between PHDI and MASLD.

To better understand the relationship between the PHDI and the risk of MASLD, we conducted RCS analysis, revealing a non-linear association. After adjusting for key covariates in Model 3, clear non-linear trends were observed (non-linear *p* = 0.002) (Figure 2). Threshold analysis indicated an inflection point at 58.073. When the PHDI exceeds this point, each unit increase in PHDI correlates with a 1.7% reduction in MASLD risk (OR = 0.983, 95% CI: 0.980–0.987, *p* < 0.001). Conversely, when the PHDI is below 58.073, each unit increase does not significantly impact MASLD risk (OR = 1.006, 95% CI: 0.997–1.016, *p* = 0.190) (Table 3). These findings underline the potential clinical utility of targeting PHDI values above 58.073 to optimize MASLD prevention, highlighting the importance of dietary interventions in reducing MASLD risk.

**Table 3:** The threshold effect of PHDI on MASLD using a two-stage phased regression model.

Models	Adjusted OR (95% CI)	*p* value
Model I		
logistic regression (the standard linear model)	0.988 (0.985–0.990)	<0.001
Model II		
Inflection point	58.073	
<58.073	1.006 (0.997–1.016)	0.190
>58.073	0.983 (0.980–0.987)	<0.001
*P* for likelihood ratio test		<0.001

Additionally, we examined the non-linear relationships between PHDI components and MASLD risk, finding significant associations with non-starchy vegetables (non-linear *p* = 0.003), whole fruits (excludes fruit juice) (non-linear *p* = 0.002), red and processed meats (non-linear *p* = 0.021), soy products (non-linear *p* = 0.006), unsaturated fatty acids (non-linear *p* < 0.001), saturated fatty acids (non-linear *p* < 0.001), and added sugar (non-linear *p* < 0.001) (Supplementary Figure S1). Threshold analyses of these components are detailed in Supplementary Table S4.

### 3.4 Subgroup analysis

**Figure 3:**
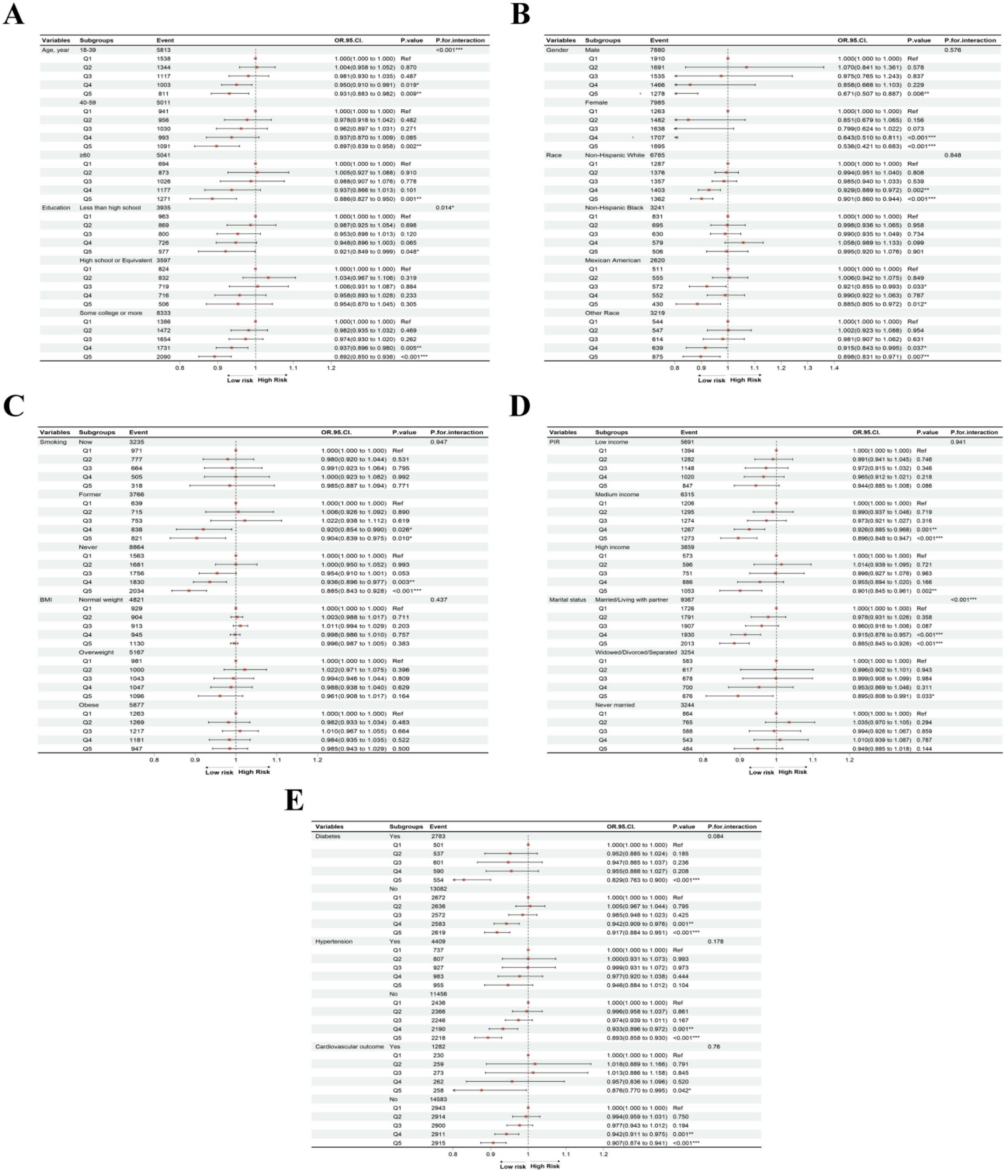
Subgroup analyses of association between PHDI and MASLD in **(A)** age and education, **(B)** gender and race, **(C)** smoking status and BMI, **(D)** PIR and marital status, **(E)** diabetes, hypertension, and cardiovascular outcome. “*”, *p* < 0.05; “**”, *p* < 0.01; “***”, *p* < 0.001.

To further investigate the association between PHDI and MASLD across diverse populations, we stratified the MASLD cohort based on age, gender, race, education level, PIR, marital status, smoking status, BMI, diabetes, hypertension, and cardiovascular outcomes. After adjusting for key variables (Model 3), stratified analysis indicated that a higher PHDI quintile was significantly associated with a reduced MASLD risk among participants identified as non-Hispanic White (*p* < 0.001), Mexican American (*p* = 0.012), or other races (*p* = 0.007); former (*p* = 0.010) or never smokers (*p* < 0.001); individuals with at least a college education (*p* < 0.001) or less than high school education (*p* = 0.048); those with median (*p* < 0.001) or high income (*p* = 0.002); those who were married/living with a partner (*p* < 0.001) or widowed/divorced/separated (*p* = 0.033); and participants without hypertension (*p* < 0.001) (Figure 3). Notably, individuals across all age groups (all *p* < 0.05), genders (all *p* < 0.001), and those with or without diabetes (all *p* < 0.001) or cardiovascular outcomes (all *p* < 0.05) benefited from increases in PHDI. Additionally, interaction effects were observed between PHDI and MASLD for gender (*p* < 0.001), education level (*p* = 0.014), and marital status (*p* < 0.001). These results suggest that females, individuals with higher education levels, and those married/living with a partner may be more sensitive to the MASLD risk reduction associated with higher PHDI.

### 3.5 WQS and mediation analysis

**Figure 4:**
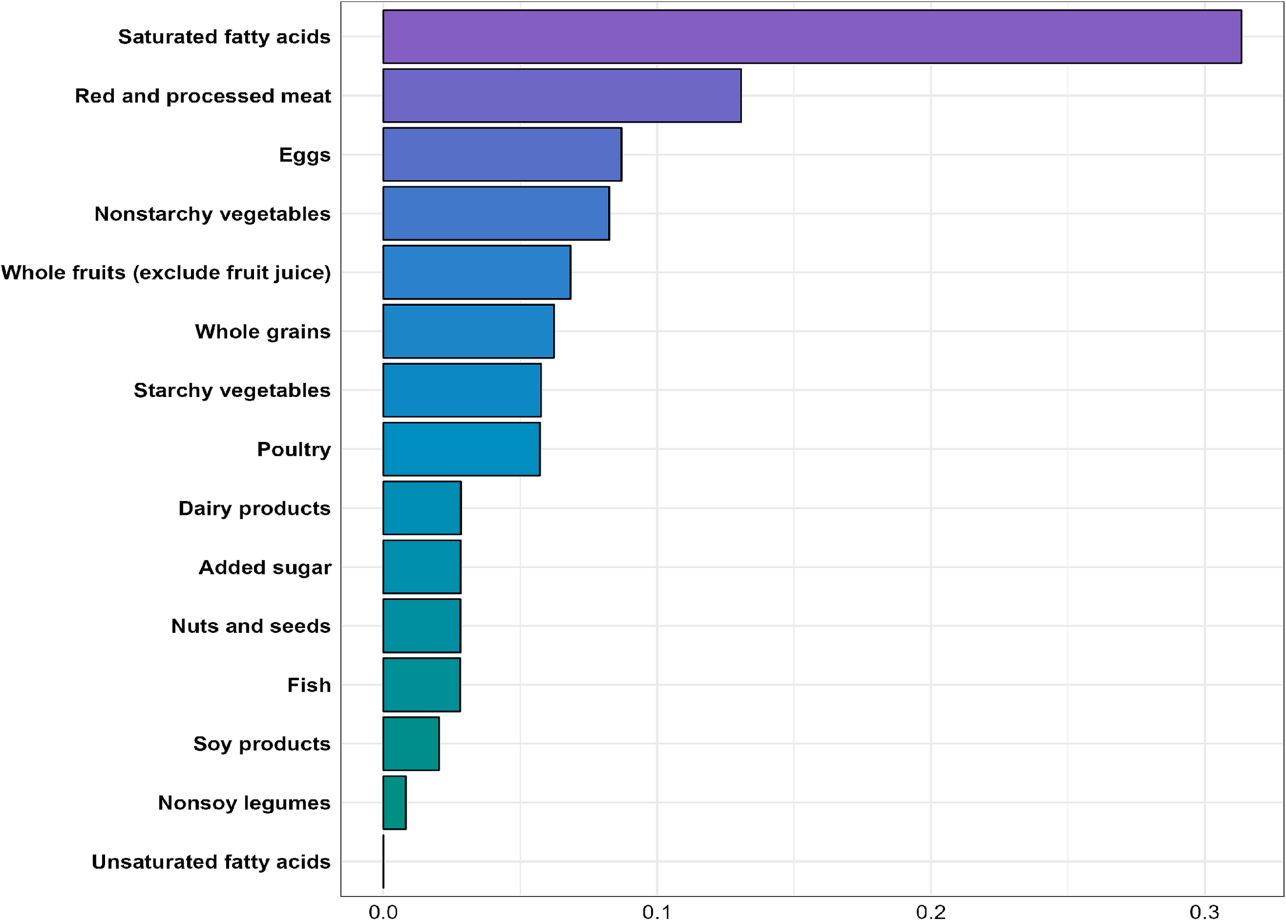
WQS analysis of PHDI components and MASLD.

The WQS regression analysis indicated an inverse association between the WQS index and MASLD risk (OR = 0.394, 95% CI: 0.318–0.487, *p* < 0.001). As shown in Figure 4, nearly all nutrient scores were inversely related to MASLD, with saturated fatty acids (weight = 0.313) identified as the most significant contributor to MASLD risk, followed by red and processed meats and eggs (weights = 0.131 and 0.087, respectively).

**Figure 5:**
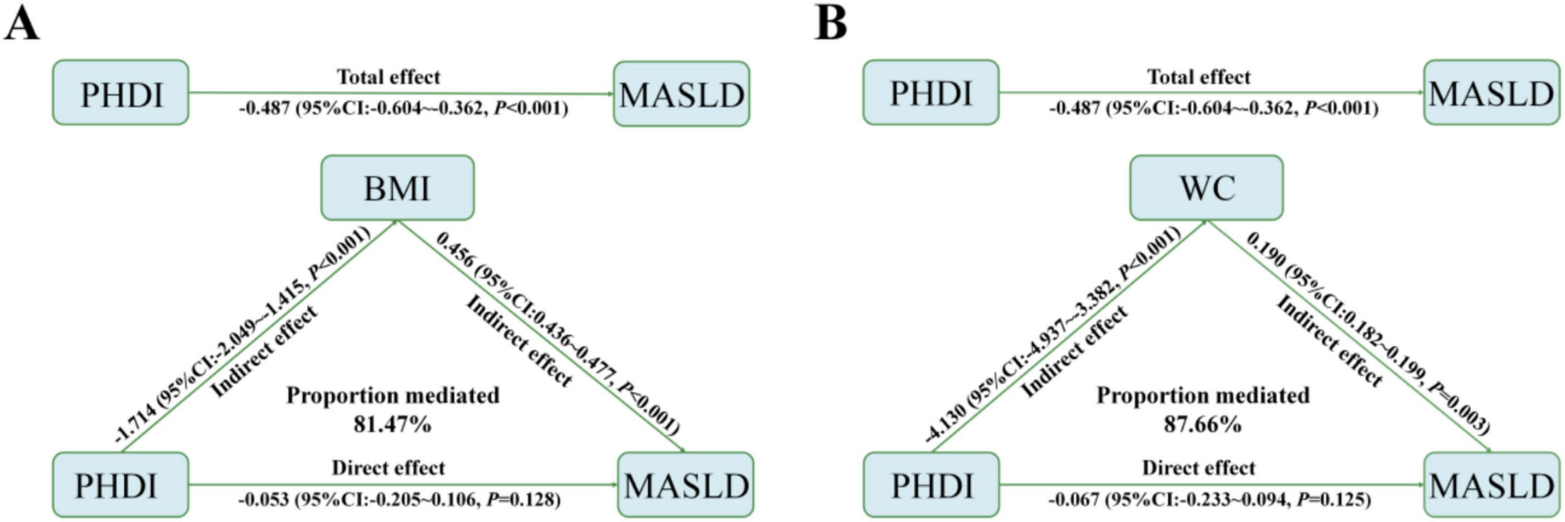
Median analyses of BMI **(A)** and WC **(B)** in the relationship between PHDI and MASLD.

Mediation analysis was conducted to explore the mediating effects of BMI and WC on the relationship between PHDI and MASLD. Specifically, the highest PHDI quintile was inversely associated with BMI (estimate = −1.714, 95% CI: −2.049 to −1.415, *p* < 0.001) and WC (estimate = −4.130, 95% CI: −4.937 to −3.382, *p* < 0.001). Both BMI and WC were positively associated with MASLD risk (OR = 0.456, 95% CI: 0.436–0.477, *p* < 0.001; OR = 0.190, 95% CI: 0.182–0.199, *p* = 0.003, respectively) (Figure 5). Ultimately, 81.47 and 87.66% of the association between the highest PHDI quintile and MASLD risk was mediated by BMI and WC, respectively. Similar results were obtained when PHDI was analyzed as a continuous variable (Supplementary Figure S2), underscoring the critical roles of these indicators in MASLD risk.

### 3.6 Sensitivity analysis

To robustly validate our findings, we conducted a series of sensitivity analyses. First, a consistent association between PHDI and MASLD was observed in earlier NHANES cycles from 2011 to 2018 (OR_Q5 vs. Q1_ = 0.565, 95% CI 0.436–0.731, *p* < 0.001, Supplementary Table S5). Second, analysis of unweighted data confirmed a significant association between PHDI and MASLD (OR_Q5 vs. Q1_ = 0.617, 95% CI 0.549–0.694, *p* < 0.001, Supplementary Table S6). Third, excluding participants with missing covariate data rather than using imputation yielded similar results (OR_Q5 vs. Q1_ = 0.564, 95% CI 0.457–0.697, *p* < 0.001, Supplementary Table S7). Finally, the inverse association between PHDI and MASLD persisted when we considered only the dietary data from the first day (OR_Q5 vs. Q1_ = 0.540, 95% CI 0.439–0.663, *p* < 0.001, Supplementary Table S8).

## 4 Discussion

In this study, we identified a significant inverse relationship between levels of the PHDI and the risk of MASLD. Participants in the highest quintile of PHDI scores exhibited an approximately 39% reduction in MASLD risk compared to those in the lowest quintile. We also observed a non-linear relationship between PHDI and MASLD, with an inflection point around 58.073. Notably, individuals who were women, had higher education levels, or were married/living with a partner showed an even greater reduction in MASLD risk with increased PHDI. These findings remained robust under various sensitivity analyses, highlighting the potential of adhering to a high PHDI as a strategy to mitigate MASLD risk.

MASLD is the most prevalent chronic liver disease, posing a significant burden due to its hepatic and extrahepatic complications. Lifestyle modifications—particularly dietary interventions—remain central to MASLD management and the improvement of insulin sensitivity (31). Prior research has established that adherence to the Mediterranean diet can substantially lower MASLD risk and its associated complications (15, 32). Similarly, plant-based diets have demonstrated protective effects against MASLD, whereas high-fat diets have been linked to its development (17, 33). Consistent with these reports, our study suggests that a higher PHDI, reflecting increased intake of plant-based and nutrient-dense foods, is linked to a reduced MASLD risk.

Beyond individual health benefits, prior investigations indicate that diets aligned with planetary health principles can reduce mortality and lower incidences of cardiovascular disease, cancer, and diabetes (34–37). In parallel, they also confer environmental co-benefits, such as reduced greenhouse gas emissions (GHGE), when compared with other healthy eating indices (e.g., HEI-2015 or DASHI) (38, 39). Adopting the planetary health diet in all counties may decrease the global water footprint by 12% (39). Our results, demonstrating an inverse association between PHDI and MASLD risk, further support the notion that planetary health-oriented diets benefit both public health and ecological sustainability.

In the present study, PHDI is composed of the scores of 15 food components categorized as adequacy (higher intake yields higher scores) and moderation (lower intake yields higher scores). A deeper look at each component showed that elevated quintiles of whole grains, non-starchy vegetables, whole fruits (excluding fruit juice), nuts and seeds, and soy products were associated with lower MASLD risk. Conversely, higher intake of saturated fatty acids correlated significantly with increased MASLD risk, aligning with prior literature (40–42).

Interestingly, a higher score for unsaturated fatty acids—an adequacy component of the PHDI—was associated with an increased risk of MASLD. The role of unsaturated fatty acids, which include monounsaturated fatty acids (MUFAs) and polyunsaturated fatty acids (PUFAs), in MASLD remains controversial. While MUFAs may reduce saturated fatty acid-induced lipotoxicity in hepatocytes (43), a diet rich in MUFAs can increase the incidence of macrovesicular steatosis and hepatic triglyceride levels, thereby elevating the risk of MASLD (44). PUFAs, on the other hand, may exacerbate cholesterol-induced mitochondrial damage hepatocytes (45), and intake of PUFAs could contribute to increased BMI, insulin resistance, and the expression of lipogenesis-related genes in the liver (46). Our findings support the harmful effect of high dietary intake of unsaturated fatty acids, as the highest quintile of unsaturated fatty acid scores was associated with a 63.8% increased in MASLD risk. Additionally, potential confounders, such as physical activity and other metabolic factors, may have influenced the observed association. Further research is needed to elucidate the specific roles of different types of unsaturated fatty acids in MASLD pathogenesis.

In our WQS regression analysis, we found that among the 15 components of the PHDI, saturated fatty acids had the strongest association with MASLD development, with a weight of 0.313—significantly higher than that of other components, consistent with previous studies (47–49). The most common saturated fatty acids include palmitic acid, lauric acid, myristic acid, and stearic acid (50), which play contradictory roles in MASLD. Excessive dietary intake of palmitic acid and lauric acid may promote insulin resistance (51), which is also correlated with the consumption of added sugar (52), contributing to hepatic steatosis and inflammation (53). Additionally, palmitic acid can enhance the expression of various inflammatory mediators, such as interleukin-32 (IL-32) and chemokine CCL20, which are involved in chronic liver inflammation (54). In contrast, dietary stearic acid may offer protective effects for hepatocytes and attenuates liver injury (55, 56). In our study, reducing the dietary intake of saturated fatty acids resulted in the most significant decrease in MASLD risk in our study, with a 47.5% reduction observed in the highest quintile of saturated fatty acid scores compared to the lowest quintile. Furthermore, added sugar was found to have a similar contribution to MASLD risk as nuts, seeds, and fish. This may be attributed to the complexity of dietary patterns, in which multiple food components interact, leading to individual components showing similar associations with MASLD risk.

Furthermore, mediation analysis indicated that the effect of PHDI on MASLD risk was largely mediated by BMI and WC, both of which are well-established independent risk factors for MASLD (57–59). Increased BMI is associated with elevated blood lipid levels, such as TG and low-density lipoprotein cholesterol (LDL-C), which mediate the relationship between BMI and MASLD by promoting lipid deposition in hepatocytes (58). Similarly, women with a WC greater than 78.5 cm had a 1.54-fold increased risk of MASLD compared to those with a WC below 78.5 cm, while men with a WC greater than 81 cm had a 1.44-fold increased risk compared to those with a WC below 81 cm (59). Our findings also suggest that a higher PHDI value was associated with lower BMI and WC levels, thereby contributing to a reduced MASLD risk. These findings may offer insights into the mechanism by which PHDI influences MASLD.

From a public health perspective, integrating PHDI-based guidelines into existing dietary recommendations could offer both metabolic and environmental advantages. Strategies might include reducing intakes of saturated fatty acids, red and processed meats, and added sugars, while prioritizing whole grains, legumes, vegetables, fruits, and other nutrient-dense, plant-based foods. Such guidelines could be reinforced by policies that promote sustainable farming practices, reduce food waste, and increase the availability of locally sourced plant-based foods. By aligning individual health goals with global sustainability objectives, PHDI-oriented interventions hold promise for dual benefits at both the population and planetary levels.

### 4.1 Strengths and limitations

A major strength of this study is its large sample size, enabling comprehensive adjustment for multiple potential confounders in assessing the relationship between PHDI and MASLD. Additionally, our various sensitivity analyses support the robustness of the findings. However, several limitations should be acknowledged. First, MASLD was identified using the Fatty Liver Index (FLI), which, while practical for large-scale research, is not as definitive as liver biopsy. Second, the cross-sectional nature of our design precludes causal inferences, and unmeasured factors such as other dietary behaviors or genetic predisposition may still confound our results. Third, relying on only 2 days of 24-h dietary recall may not fully represent habitual long-term intake. Lastly, the NHANES dataset focuses on the U.S. population; thus, our findings might not be fully generalizable to regions with different dietary patterns, food availability, and cultural practices. Future longitudinal or interventional studies in diverse populations are warranted to confirm the applicability of the PHDI and to further elucidate the causal mechanisms linking dietary patterns to MASLD risk.

## 5 Conclusion

In summary, this study demonstrates that adherence to the PHDI is associated with a reduced risk of MASLD, with BMI and WC acting as partial mediators. Saturated fatty acids emerged as the most influential component in PHDI’s impact on MASLD risk. These findings provide valuable insights for MASLD management and support efforts toward environmental sustainability. Further prospective and intervention studies are warranted to validate these results. Incorporating PHDI-based recommendations into national dietary guidelines could play a crucial role in MASLD prevention and support broader sustainability initiatives.

## Data Availability

Publicly available datasets were analyzed in this study. This data can be found at: https://www.cdc.gov/nchs/nhanes/index.htm.

## Footnotes

^1^ https://www.r-project.org/

## Glossary

MASLD: Metabolic Dysfunction-Associated Steatotic Liver Disease
NAFLD: Nonalcoholic Fatty Liver Disease
MASH: Metabolic Dysfunction-Associated Steatohepatitis
HCC: Hepatocellular Carcinoma
PHDI: Planetary Health Diet Index
MD: Mediterranean Diet
PBDs: Plant-Based Diets
HEI: Healthy Eating Index
NCHS: National Center for Health Statistics
FLI: Fatty Liver Index
USDA: United States Department of Agriculture
FPED: Food Patterns Equivalent Database
SLD: Steatotic Liver Disease
TG: Triglyceride
BMI: Body Mass Index
GGT: Gamma-Glutamyl Transferase
WC: Waist Circumference
PIR: Poverty Income Ratio
ALT: Alanine Aminotransferase
AST: Aspartate Aminotransferase
OR: Odds Ratio
CI: Confidence Intervals
RCS: Restricted Cubic Spline
WQS: Weighted Quantile Sum
HEI-2015: Healthy Eating Index-2015
DASHI: Dietary Approaches to Stop Hypertension
AHEI: Alternative Healthy Eating Index
AMED: Alternate Mediterranean Diet
MUFAs: Monounsaturated Fatty Acids
PUFAs: Polyunsaturated Fatty Acids
GHGE: Greenhouse Gas Emissions
IL-32: Interleukin-32
LDL-C: Low-Density Lipoprotein Cholesterol
