# Supplementary material for: Adherence to the planetary health diet index and metabolic dysfunction-associated steatotic liver disease: a cross-sectional study
Source: Front Nutr. 2025 Feb 20;12:1534604. doi: 10.3389/fnut.2025.1534604 (PMC11882404; doi:10.3389/fnut.2025.1534604)
Supplement: Supplementary file 1 [file Table_1.docx]

| **Supplementary Table S1 Baseline characteristics for MASLD or non-MASLD individuals** | | | | |
| --- | --- | --- | --- | --- |
| **Characteristics** | **Overall**, N = 15865 (100%)^1^ | **Non-MASLD**, N = 9740 (62%)^1^ | **MASLD**, N = 6125 (38%)^1^ | **P Value**^2^ |
| Age |  |  |  | <0.001^***^ |
| 18-39 years | 5,813 (39.22%) | 4,118 (44.51%) | 1,695 (30.54%) |  |
| 40-59 years | 5,011 (35.64%) | 2,819 (32.44%) | 2,192 (40.90%) |  |
| >=60 years | 5,041 (25.14%) | 2,803 (23.05%) | 2,238 (28.56%) |  |
| Gender |  |  |  | <0.001^***^ |
| Male | 7,880 (49.57%) | 4,703 (46.86%) | 3,177 (54.01%) |  |
| Female | 7,985 (50.43%) | 5,037 (53.14%) | 2,948 (45.99%) |  |
| Race |  |  |  | <0.001^***^ |
| Non-Hispanic White | 6,785 (66.65%) | 4,143 (67.01%) | 2,642 (66.07%) |  |
| Non-Hispanic Black | 3,241 (11%) | 1,977 (10.88%) | 1,264 (11.20%) |  |
| Mexican American | 2,620 (8.95%) | 1,433 (7.80%) | 1,187 (10.85%) |  |
| Other Race | 3,219 (13.39%) | 2,187 (14.31%) | 1,032 (11.88%) |  |
| Marital status |  |  |  | <0.001^***^ |
| Married/Living with partner | 9,380 (62.33%) | 5,617 (60.57%) | 3,763 (65.20%) |  |
| Never married | 3,228 (19.78%) | 2,263 (22.47%) | 965 (15.36%) |  |
| Widowed/Divorced/  Separated | 3,257 (17.89%) | 1,860 (16.96%) | 1,397 (19.43%) |  |
| Education |  |  |  | <0.001^***^ |
| High school or equivalent | 3,589 (23.26%) | 2,127 (22.06%) | 1,462 (25.21%) |  |
| Less than high school | 3,945 (16.78%) | 2,216 (15.27%) | 1,729 (19.25%) |  |
| Some college or more | 8,331 (59.97%) | 5,397 (62.67%) | 2,934 (55.54%) |  |
| PIR |  |  |  | 0.408 |
| High income | 3,835 (34.08%) | 2,477 (34.66%) | 1,358 (33.12%) |  |
| Low income | 5,758 (27.21%) | 3,424 (26.62%) | 2,334 (28.17%) |  |
| Medium income | 6,272 (38.72%) | 3,839 (38.72%) | 2,433 (38.70%) |  |
| Smoking |  |  |  | <0.001^***^ |
| Now | 3,239 (21.17%) | 2,110 (22.04%) | 1,129 (19.73%) |  |
| Former | 3,745 (24.05%) | 2,032 (21.86%) | 1,713 (27.65%) |  |
| Never | 8,881 (54.78%) | 5,598 (56.10%) | 3,283 (52.62%) |  |
| Diabetes |  |  |  | <0.001^***^ |
| No | 13,082 (87.04%) | 8,767 (93.38%) | 4,315 (76.63%) |  |
| Yes | 2,783 (12.96%) | 973 (6.62%) | 1,810 (23.37%) |  |
|  |  |  |  |  |

| **Supplementary Table 1 Baseline characteristics for MASLD or non-MASLD individuals** | | | | |
| --- | --- | --- | --- | --- |
| **Characteristics** | **Overall**  N = 15865 (100%)^1^ | **Non-MASLD**  N = 9740 (62%)^1^ | **MASLD**  N = 6125 (38%)^1^ | **P Value**^2^ |
| Hypertension |  |  |  | <0.001^***^ |
| No | 11,456 (75.41%) | 7,759 (82.87%) | 3,697 (63.19%) |  |
| Yes | 4,409 (24.59%) | 1,981 (17.13%) | 2,428 (36.81%) |  |
| Cardiovascular outcome |  |  |  | <0.001^***^ |
| No | 14,583 (93.19%) | 9,174 (95.09%) | 5,409 (90.07%) |  |
| Yes | 1,282 (6.81%) | 566 (4.91%) | 716 (9.93%) |  |
| ALT | 21 (16, 28) | 19 (15, 25) | 25 (19, 33) | <0.001^***^ |
| AST | 22 (19, 27) | 22 (19, 26) | 23 (19, 28) | <0.001^***^ |
| GGT | 19 (14, 29) | 16 (12, 23) | 25 (18, 38) | <0.001^***^ |
| ALP | 65 (53, 79) | 62 (51, 76) | 70 (57, 84) | <0.001^***^ |
| LYM | 1.90 (1.60, 2.40) | 1.90 (1.50, 2.30) | 2.00 (1.70, 2.50) | <0.001^***^ |
| NEU | 3.70 (2.90, 4.80) | 3.50 (2.70, 4.50) | 4.10 (3.30, 5.20) | <0.001^***^ |
| Albumin | 4.30 (4.00, 4.50) | 4.30 (4.10, 4.50) | 4.20 (4.00, 4.40) | <0.001^***^ |
| BMI | 27.67 (23.90, 32.40) | 24.90 (22.46, 27.60) | 33.48 (30.39, 37.71) | <0.001^***^ |
| Total Cholesterol | 189 (164, 217) | 186 (161, 214) | 194 (168, 223) | <0.001^***^ |
| Triglyceride | 1.16 (0.80, 1.72) | 0.97 (0.70, 1.34) | 1.61 (1.14, 2.27) | <0.001^***^ |
| PHDI | 67.02 (57.70, 77.29) | 68.09 (58.23, 78.65) | 65.61 (56.91, 74.83) | <0.001^***^ |
| PHDI (Quintile) |  |  |  | <0.001^***^ |
| Q1 | 3,173(19.55%) | 1,876(18.59%) | 1,297(21.13%) |  |
| Q2 | 3,173(20.39%) | 1,856(19.39%) | 1,317(22.04%) |  |
| Q3 | 3,173(19.44%) | 1,895(18.36%) | 1,278(21.21%) |  |
| Q4 | 3,173(20.12%) | 1,938(20.56%) | 1,235(19.40%) |  |
| Q5 | 3,173(20.50%) | 2,175(23.10%) | 998(16.22%) |  |
| ^1^n (unweighted) (%); Median (IQR) | | | | |
| ^2^chi-squared test with Rao & Scott's second-order correction; Wilcoxon rank-sum test for complex survey  samples | | | | |
| “*”: P<0.05; “**”: P<0.01;“***”: P<0.001. | | | | |
