# Supplementary material for: Adherence to the planetary health diet index and metabolic dysfunction-associated steatotic liver disease: a cross-sectional study
Source: Front Nutr. 2025 Feb 20;12:1534604. doi: 10.3389/fnut.2025.1534604 (PMC11882404; doi:10.3389/fnut.2025.1534604)
Supplement: Supplementary file 2 [file Table_2.docx]

| Supplementary Table S2 The associations between PHDI, other dietary index, and MASLD | | | | | | |
| --- | --- | --- | --- | --- | --- | --- |
| Characteristic | Model 1 | | Model 2 | | Model 3 | |
|  | OR (95% CI) | *P* value | OR (95% CI) | *P* value | OR (95% CI) | *P* value |
| PHDI | 0.985 (0.982, 0.989) | <0.001^***^ | 0.983 (0.979, 0.986) | <0.001^***^ | 0.986 (0.982, 0.990) | <0.001^***^ |
| PHDI (Quintile) | | | | | | |
| Q1 | Ref |  | Ref |  | Ref |  |
| Q2 | 0.970 (0.823, 1.143) | 0.710 | 0.934 (0.789, 1.107) | 0.427 | 0.944 (0.782, 1.141) | 0.550 |
| Q3 | 0.954 (0.812, 1.120) | 0.559 | 0.884 (0.752, 1.040) | 0.135 | 0.838 (0.700, 1.004) | 0.055 |
| Q4 | 0.800 (0.670, 0.955) | 0.014^*^ | 0.736 (0.617, 0.878) | 0.001^**^ | 0.712 (0.599, 0.847) | <0.001^***^ |
| Q5 | 0.569 (0.475, 0.683) | <0.001^***^ | 0.517 (0.430, 0.622) | <0.001^***^ | 0.588 (0.474, 0.728) | <0.001^***^ |
| *P* for trend | | <0.001^***^ |  | <0.001^***^ |  | <0.001^***^ |
| DASHI | 0.839 (0.800, 0.880) | <0.001^***^ | 0.829 (0.790, 0.871) | <0.001^***^ | 0.849 (0.805, 0.897) | <0.001^***^ |
| DASHI (Quintile) | | | | | | |
| Q1 | Ref |  | Ref |  | Ref |  |
| Q2 | 0.860 (0.725, 1.021) | 0.084 | 0.882 (0.743, 1.046) | 0.147 | 0.898 (0.756, 1.067) | 0.218 |
| Q3 | 0.692 (0.588, 0.815) | <0.001^***^ | 0.701 (0.595, 0.827) | <0.001^***^ | 0.740 (0.616, 0.890) | 0.002^**^ |
| Q4 | 0.563 (0.481, 0.659) | <0.001^***^ | 0.556 (0.474, 0.652) | <0.001^***^ | 0.591 (0.497, 0.703) | <0.001^***^ |
| Q5 | 0.611 (0.507, 0.736) | <0.001^***^ | 0.593 (0.491, 0.718) | <0.001^***^ | 0.639 (0.517, 0.789) | <0.001^***^ |
| *P* for trend | | <0.001^***^ |  | <0.001^***^ |  | <0.001^***^ |
| AHEI | 0.982 (0.977, 0.986) | <0.001^***^ | 0.977 (0.972, 0.981) | <0.001^***^ | 0.979 (0.973, 0.984) | <0.001^***^ |
| AHEI (Quintile) | | | | | | |
| Q1 | Ref |  | Ref |  | Ref |  |
| Q2 | 1.073 (0.868, 1.325) | 0.511 | 1.000 (0.813, 1.231) | 0.997 | 0.929 (0.747, 1.156) | 0.503 |
| Q3 | 0.928 (0.760, 1.134) | 0.463 | 0.814 (0.663, 0.999) | 0.049^*^ | 0.739 (0.593, 0.921) | 0.008^**^ |
| Q4 | 0.973 (0.826, 1.147) | 0.742 | 0.849 (0.717, 1.005) | 0.057 | 0.804 (0.657, 0.983) | 0.034^*^ |
| Q5 | 0.539 (0.448, 0.648) | <0.001^***^ | 0.455 (0.376, 0.552) | <0.001^***^ | 0.486 (0.390, 0.606) | <0.001^***^ |
| *P* for trend | | <0.001^***^ |  | <0.001^***^ |  | <0.001^***^ |
| AMED | 0.832 (0.800, 0.866) | <0.001^***^ | 0.799 (0.769, 0.831) | <0.001^***^ | 0.824 (0.787, 0.863) | <0.001^***^ |
| AMED (Quintile) | | | | | | |
| Q1 | Ref |  | Ref |  | Ref |  |
| Q2 | 1.013 (0.839, 1.222) | 0.895 | 0.942 (0.785, 1.132) | 0.520 | 0.875 (0.720, 1.064) | 0.178 |
| Q3 | 0.846 (0.705, 1.016) | 0.073 | 0.769 (0.643, 0.919) | 0.004^**^ | 0.757 (0.623, 0.921) | 0.006^**^ |
| Q4 | 0.780 (0.652, 0.934) | 0.007^**^ | 0.681 (0.570, 0.813) | <0.001^***^ | 0.656 (0.536, 0.802) | <0.001^***^ |
| Q5 | 0.488 (0.405, 0.587) | <0.001^***^ | 0.416 (0.346, 0.502) | <0.001^***^ | 0.455 (0.367, 0.563) | <0.001^***^ |
| *P* for trend |  | <0.001^***^ |  | <0.001^***^ |  | <0.001^***^ |

Abbreviation: PHDI, Planetary Health Diet Index; DASHI, Dietary Approaches to Stop Hypertension Index; AHEI, Alternative Healthy Eating Index; AMED, Alternate Mediterranean Diet Score.
“^*^”, *P*<0.05; “^**^”, *P*<0.01; “^***^”, *P*<0.001.
