# Supplementary material for: Adherence to the planetary health diet index and metabolic dysfunction-associated steatotic liver disease: a cross-sectional study
Source: Front Nutr. 2025 Feb 20;12:1534604. doi: 10.3389/fnut.2025.1534604 (PMC11882404; doi:10.3389/fnut.2025.1534604)
Supplement: Supplementary file 3 [file Table_3.docx]

| Supplementary Table S3 The associations between components of PHDI and MASLD | | | | | | |
| --- | --- | --- | --- | --- | --- | --- |
| Characteristic | Model 1 | | Model 2 | | Model 3 | |
|  | OR (95% CI) | *P* value | OR (95% CI) | *P* value | OR (95% CI) | *P* value |
| PHDI | 0.987 (0.984, 0.990) | <0.001^***^ | 0.985 (0.982, 0.988) | <0.001^***^ | 0.987 (0.983, 0.990) | <0.001^***^ |
| PHDI (Quintile) | | | | | | |
| Q1 | Ref |  | Ref |  | Ref |  |
| Q2 | 0.999 (0.865, 1.155) | 0.994 | 0.964 (0.832, 1.117) | 0.621 | 0.985 (0.834, 1.163) | 0.857 |
| Q3 | 1.016 (0.870, 1.186) | 0.839 | 0.942 (0.808, 1.098) | 0.441 | 0.903 (0.763, 1.069) | 0.233 |
| Q4 | 0.830 (0.706, 0.976) | 0.024 | 0.757 (0.646, 0.886) | 0.001^**^ | 0.745 (0.637, 0.872) | <0.001^**^ |
| Q5 | 0.617 (0.527, 0.723) | <0.001^***^ | 0.557 (0.477, 0.650) | <0.001^***^ | 0.610 (0.508, 0.733) | <0.001^***^ |
| *P* for trend | | <0.001^***^ |  | <0.001^***^ |  | <0.001^***^ |
| Whole grains | 0.978 (0.965, 0.991) | 0.001^**^ | 0.972 (0.960, 0.985) | <0.001^***^ | 0.976 (0.962, 0.989) | 0.001^**^ |
| Whole grains (Quintile) | | | | | | |
| Q1 | Ref |  | Ref |  | Ref |  |
| Q2 | 0.876 (0.744, 1.031) | 0.110 | 0.857 (0.726, 1.012) | 0.069 | 0.882 (0.738, 1.054) | 0.165 |
| Q3 | 0.836 (0.711, 0.984) | 0.031^*^ | 0.799 (0.681, 0.936) | 0.006^**^ | 0.803 (0.673, 0.957) | 0.015^*^ |
| Q4 | 0.774 (0.656, 0.913) | 0.003^**^ | 0.728 (0.620, 0.854) | <0.001^***^ | 0.767 (0.645, 0.912) | 0.003^**^ |
| Q5 | 0.835 (0.711, 0.980) | 0.027^*^ | 0.786 (0.668, 0.924) | 0.004^**^ | 0.829 (0.692, 0.993) | 0.041^*^ |
| *P* for trend | | 0.004^**^ |  | <0.001^***^ |  | 0.005^**^ |
| Starchy vegetables | 0.988 (0.963, 1.013) | 0.351 | 0.996 (0.970, 1.022) | 0.745 | 0.992 (0.964, 1.021) | 0.587 |
| Starchy vegetables (Quintile) | | | | | | |
| Q1 | Ref |  | Ref |  | Ref |  |
| Q2 | 0.916 (0.786, 1.067) | 0.255 | 0.947 (0.812, 1.105) | 0.487 | 0.921 (0.784, 1.081) | 0.311 |
| Q3 | 0.880 (0.740, 1.047) | 0.149 | 0.926 (0.777, 1.104) | 0.386 | 0.920 (0.772, 1.095) | 0.343 |
| Q4 | 0.842 (0.709, 1.000) | 0.050 | 0.890 (0.746, 1.061) | 0.192 | 0.918 (0.768, 1.098) | 0.345 |
| Q5 | 1.013 (0.841, 1.221) | 0.888 | 1.063 (0.875, 1.292) | 0.534 | 1.065 (0.870, 1.304) | 0.539 |
| *P* for trend | | 0.745 |  | 0.795 |  | 0.601 |
| Nonstarchy vegetables | 0.925 (0.895, 0.956) | <0.001^***^ | 0.917 (0.887, 0.948) | <0.001^***^ | 0.923 (0.889, 0.958) | <0.001^***^ |
| Nonstarchy vegetables (Quintile) | | | | | | |
| Q1 | Ref |  | Ref |  | Ref |  |
| Q2 | 0.855 (0.724, 1.010) | 0.064 | 0.854 (0.721, 1.011) | 0.067 | 0.810 (0.673, 0.975) | 0.027^*^ |
| Q3 | 0.820 (0.693, 0.970) | 0.021^*^ | 0.798 (0.675, 0.944) | 0.009^**^ | 0.753 (0.631, 0.898) | 0.002^**^ |
| Q4 | 0.849 (0.737, 0.978) | 0.023^*^ | 0.824 (0.714, 0.951) | 0.009^**^ | 0.773 (0.665, 0.899) | 0.001^**^ |
| Q5 | 0.690 (0.581, 0.821) | <0.001^***^ | 0.660 (0.555, 0.786) | <0.001^***^ | 0.669 (0.554, 0.808) | <0.001^***^ |
| *P* for trend | <0.001^***^ |  | <0.001^***^ |  | <0.001^***^ |  |

| Supplementary Table 2 continued | | | | | | |
| --- | --- | --- | --- | --- | --- | --- |
| Characteristic | Model 1 | | Model 2 | | Model 3 | |
|  | OR (95% CI) | P value | OR (95% CI) | P value | OR (95% CI) | P value |

| Whole fruits (exclude fruit juice) | 0.977 (0.962, 0.991) | 0.002^**^ | 0.963 (0.949, 0.977) | <0.001^***^ | 0.972 (0.957, 0.987) | <0.001^***^ |
| --- | --- | --- | --- | --- | --- | --- |
| Whole fruits (exclude fruit juice) (Quintile) | | | | | | |

| Q1 | Ref |  | Ref |  | Ref |  |
| --- | --- | --- | --- | --- | --- | --- |
| Q2 | 0.977 (0.846, 1.129) | 0.750 | 0.936 (0.806, 1.088) | 0.388 | 0.924 (0.783, 1.090) | 0.345 |
| Q3 | 0.889 (0.748, 1.056) | 0.179 | 0.817 (0.687, 0.972) | 0.023^*^ | 0.814 (0.674, 0.982) | 0.032^*^ |
| Q4 | 0.874 (0.757, 1.010) | 0.068 | 0.787 (0.681, 0.910) | 0.001^**^ | 0.814 (0.700, 0.946) | 0.008^**^ |
| Q5 | 0.804 (0.695, 0.931) | 0.004^**^ | 0.700 (0.606, 0.808) | <0.001^***^ | 0.755 (0.652, 0.875) | <0.001^***^ |
| *P* for trend |  | 0.002^**^ |  | <0.001^***^ |  | <0.001^***^ |
| Dairy products | 0.996 (0.977, 1.015) | 0.680 | 0.992 (0.973, 1.012) | 0.431 | 0.981 (0.960, 1.002) | 0.075 |
| Dairy products (Quintile) | | | | | | |
| Q1 | Ref |  | Ref |  | Ref |  |
| Q2 | 0.979 (0.840, 1.143) | 0.790 | 0.971 (0.832, 1.132) | 0.700 | 0.864 (0.735, 1.016) | 0.076 |
| Q3 | 0.984 (0.830, 1.166) | 0.849 | 0.957 (0.805, 1.137) | 0.613 | 0.887 (0.734, 1.072) | 0.210 |
| Q4 | 0.985 (0.840, 1.156) | 0.854 | 0.967 (0.822, 1.137) | 0.682 | 0.844 (0.715, 0.996) | 0.045 |
| Q5 | 1.027 (0.850, 1.242) | 0.779 | 0.997 (0.821, 1.212) | 0.978 | 0.905 (0.730, 1.121) | 0.355 |
| P for trend |  | 0.815 |  | 0.919 |  | 0.285 |
| Red and processed meat | 0.959 (0.945, 0.974) | <0.001^***^ | 0.964 (0.949, 0.979) | <0.001^***^ | 0.973 (0.957, 0.990) | 0.002^**^ |
| Red and processed meat (Quintile) | | | | | | |
| Q1 | Ref |  | Ref |  | Ref |  |
| Q2 | 0.775 (0.639, 0.942) | 0.011^*^ | 0.808 (0.664, 0.982) | 0.032^*^ | 0.791 (0.635, 0.985) | 0.036^*^ |
| Q3 | 0.675 (0.542, 0.839) | 0.001^**^ | 0.685 (0.552, 0.851) | 0.001^**^ | 0.738 (0.588, 0.926) | 0.009^**^ |
| Q4 | 0.647 (0.530, 0.790) | <0.001^***^ | 0.678 (0.555, 0.828) | <0.001^***^ | 0.775 (0.635, 0.946) | 0.013^*^ |
| Q5 | 0.697 (0.560, 0.869) | 0.002^**^ | 0.737 (0.591, 0.919) | 0.007^**^ | 0.792 (0.607, 1.033) | 0.084 |
| *P* for trend |  | <0.001^***^ |  | <0.001^***^ |  | 0.002^**^ |
| Poultry | 1.000 (0.990, 1.010) | 0.981 | 0.998 (0.988, 1.008) | 0.664 | 1.002 (0.991, 1.012) | 0.762 |
| Poultry (Quintile) | | | | | | |
| Q1 | Ref |  | Ref |  | Ref |  |
| Q2 | 0.918 (0.779, 1.082) | 0.304 | 0.917 (0.775, 1.086) | 0.313 | 0.959 (0.795, 1.158) | 0.661 |
| Q3 | 1.020 (0.870, 1.194) | 0.809 | 1.011 (0.859, 1.191) | 0.892 | 1.068 (0.902, 1.263) | 0.442 |
| Q4 | 0.923 (0.777, 1.097) | 0.362 | 0.904 (0.769, 1.064) | 0.223 | 0.931 (0.789, 1.099) | 0.393 |
| Q5 | 1.087 (0.939, 1.258) | 0.261 | 1.052 (0.904, 1.224) | 0.512 | 1.112 (0.941, 1.315) | 0.208 |
| P for trend |  | 0.650 |  | 0.978 |  | 0.434 |

| Supplementary Table 2 continued | | | | | | |
| --- | --- | --- | --- | --- | --- | --- |
| Characteristic | Model 1 | | Model 2 | | Model 3 | |
|  | OR (95% CI) | *P* value | OR (95% CI) | *P* value | OR (95% CI) | *P* value |
| Eggs | 0.982 (0.970, 0.995) | 0.006^**^ | 0.989 (0.977, 1.001) | 0.075 | 0.993 (0.980, 1.006) | 0.260 |
| Eggs (Quintile) | | | | | | |
| Q1 | Ref |  | Ref |  | Ref |  |
| Q2 | 0.888 (0.758, 1.041) | 0.141 | 0.897 (0.764, 1.052) | 0.178 | 0.935 (0.777, 1.124) | 0.469 |
| Q3 | 0.793 (0.700, 0.899) | <0.001^***^ | 0.852 (0.751, 0.967) | 0.013^*^ | 0.890 (0.780, 1.015) | 0.082 |
| Q4 | 0.834 (0.695, 1.001) | 0.052 | 0.893 (0.749, 1.064) | 0.203 | 0.908 (0.756, 1.090) | 0.298 |
| Q5 | 0.884 (0.741, 1.054) | 0.169 | 0.926 (0.777, 1.105) | 0.392 | 0.961 (0.795, 1.161) | 0.677 |
| *P* for trend |  | 0.131 |  | 0.438 |  | 0.606 |
| Fish | 0.992 (0.980, 1.004) | 0.183 | 0.988 (0.976, 1.000) | 0.059 | 0.988 (0.974, 1.002) | 0.092 |
| Fish (Quintile) | | | | | | |
| Q1 | Ref |  | Ref |  | Ref |  |
| Q2 | 0.918 (0.770, 1.094) | 0.334 | 0.899 (0.754, 1.070) | 0.228 | 0.908 (0.743, 1.110) | 0.343 |
| Q3 | 0.874 (0.719, 1.062) | 0.175 | 0.843 (0.690, 1.030) | 0.094 | 0.811 (0.655, 1.004) | 0.054 |
| Q4 | 0.802 (0.584, 1.103) | 0.173 | 0.752 (0.550, 1.029) | 0.074 | 0.776 (0.563, 1.070) | 0.121 |
| Q5 | 1.093 (0.926, 1.289) | 0.290 | 1.073 (0.893, 1.288) | 0.448 | 1.082 (0.869, 1.347) | 0.478 |
| *P* for trend |  | 0.443 |  | 0.221 |  | 0.314 |
| Nuts and seeds | 0.974 (0.963, 0.985) | <0.001^***^ | 0.968 (0.957, 0.980) | <0.001^***^ | 0.975 (0.963, 0.987) | <0.001^***^ |
| Nuts and seeds (Quintile) | | | | | | |
| Q1 | Ref |  | Ref |  | Ref |  |
| Q2 | 0.858 (0.733, 1.005) | 0.057 | 0.852 (0.728, 0.996) | 0.044^*^ | 0.897 (0.755, 1.067) | 0.217 |
| Q3 | 0.829 (0.718, 0.958) | 0.012^*^ | 0.800 (0.689, 0.928) | 0.004^**^ | 0.828 (0.712, 0.963) | 0.015^*^ |
| Q4 | 0.706 (0.591, 0.843) | <0.001^***^ | 0.665 (0.557, 0.794) | <0.001^***^ | 0.727 (0.604, 0.876) | 0.001^**^ |
| Q5 | 0.744 (0.619, 0.896) | 0.002^**^ | 0.703 (0.578, 0.856) | 0.001^**^ | 0.760 (0.611, 0.945) | 0.014^*^ |
| *P* for trend |  | <0.001^***^ |  | <0.001^***^ |  | <0.001^***^ |
| Nonsoy legumes | 1.022 (0.996, 1.050) | 0.099 | 1.014 (0.986, 1.042) | 0.334 | 1.011 (0.981, 1.043) | 0.469 |
| Nonsoy legumes (Quintile) | | | | | | |
| Q1 | Ref |  | Ref |  | Ref |  |
| Q2 | 0.949 (0.780, 1.155) | 0.600 | 0.979 (0.800, 1.200) | 0.839 | 0.984 (0.806, 1.200) | 0.869 |
| Q3 | 1.012 (0.842, 1.218) | 0.894 | 0.992 (0.819, 1.202) | 0.935 | 0.999 (0.806, 1.239) | 0.996 |
| Q4 | 1.173 (0.965, 1.427) | 0.108 | 1.117 (0.925, 1.348) | 0.247 | 1.116 (0.920, 1.356) | 0.262 |
| Q5 | 1.081 (0.869, 1.345) | 0.479 | 1.031 (0.812, 1.309) | 0.802 | 1.001 (0.770, 1.301) | 0.992 |
| *P* for trend |  | 0.192 |  | 0.507 |  | 0.658 |

| Supplementary Table 2 continued | | | | | | |
| --- | --- | --- | --- | --- | --- | --- |
| Characteristic | Model 1 | | Model 2 | | Model 3 | |
|  | OR (95% CI) | *P* value | OR (95% CI) | *P* value | OR (95% CI) | *P* value |
| Soy products | 0.855 (0.816, 0.897) | <0.001^***^ | 0.872 (0.831, 0.914) | <0.001^***^ | 0.908 (0.862, 0.956) | <0.001^***^ |
| Soy products (Quintile) | | | | | | |
| Q1 | Ref |  | Ref |  | Ref |  |
| Q2 | 0.965 (0.743, 1.253) | 0.787 | 0.920 (0.699, 1.210) | 0.546 | 0.996 (0.727, 1.365) | 0.980 |
| Q3 | 0.755 (0.571, 0.998) | 0.048^*^ | 0.790 (0.597, 1.046) | 0.099 | 0.854 (0.635, 1.150) | 0.295 |
| Q4 | 0.517 (0.419, 0.639) | <0.001^***^ | 0.539 (0.435, 0.667) | <0.001^***^ | 0.595 (0.477, 0.743) | <0.001^***^ |
| Q5 | 0.523 (0.400, 0.685) | <0.001^***^ | 0.577 (0.442, 0.754) | <0.001^***^ | 0.710 (0.534, 0.944) | 0.019^*^ |
| *P* for trend |  | <0.001^***^ |  | <0.001^***^ |  | <0.001^***^ |
| Unsaturated fatty acids | 1.110 (1.077, 1.144) | <0.001^***^ | 1.104 (1.070, 1.138) | <0.001^***^ | 1.102 (1.065, 1.140) | <0.001^***^ |
| Unsaturated fatty acids (Quintile) | | | | | | |
| Q1 | Ref |  | Ref |  | Ref |  |
| Q2 | 1.102 (0.916, 1.326) | 0.300 | 1.095 (0.906, 1.323) | 0.344 | 1.127 (0.919, 1.383) | 0.246 |
| Q3 | 1.284 (1.096, 1.505) | 0.002^**^ | 1.272 (1.084, 1.494) | 0.004^**^ | 1.304 (1.095, 1.553) | 0.003^**^ |
| Q4 | 1.641 (1.391, 1.937) | <0.001^***^ | 1.599 (1.360, 1.880) | <0.001^***^ | 1.594 (1.337, 1.899) | <0.001^***^ |
| Q5 | 1.624 (1.380, 1.911) | <0.001^***^ | 1.591 (1.340, 1.889) | <0.001^***^ | 1.638 (1.356, 1.978) | <0.001^***^ |
| *P* for trend |  | <0.001^***^ |  | <0.001^***^ |  | <0.001^***^ |
| Saturated fatty acids | 0.863 (0.830, 0.899) | <0.001^***^ | 0.861 (0.826, 0.896) | <0.001^***^ | 0.866 (0.827, 0.906) | <0.001^***^ |
| Saturated fatty acids (Quintile) | | | | | | |
| Q1 | Ref |  | Ref |  | Ref |  |
| Q2 | 0.733 (0.612, 0.879) | 0.001^**^ | 0.712 (0.592, 0.858) | <0.001^***^ | 0.708 (0.572, 0.876) | 0.002^**^ |
| Q3 | 0.750 (0.618, 0.910) | 0.004^**^ | 0.745 (0.611, 0.908) | 0.004^**^ | 0.755 (0.608, 0.937) | 0.011^*^ |
| Q4 | 0.636 (0.524, 0.773) | <0.001^***^ | 0.641 (0.524, 0.782) | <0.001^***^ | 0.661 (0.536, 0.815) | <0.001^***^ |
| Q5 | 0.537 (0.439, 0.658) | <0.001^***^ | 0.521 (0.425, 0.640) | <0.001^***^ | 0.525 (0.417, 0.662) | <0.001^***^ |
| *P* for trend |  | <0.001^***^ |  | <0.001^***^ |  | <0.001^***^ |
| Added sugar | 0.867 (0.779, 0.964) | 0.009^**^ | 0.813 (0.732, 0.903) | <0.001^***^ | 0.785 (0.701, 0.878) | <0.001^***^ |
| Added sugar (Quintile) | | | | | | |
| Q1 | Ref |  | Ref |  | Ref |  |
| Q2 | 0.868 (0.726, 1.037) | 0.117 | 0.805 (0.675, 0.960) | 0.016^*^ | 0.782 (0.651, 0.939) | 0.009^**^ |
| Q3 | 0.842 (0.722, 0.982) | 0.029^*^ | 0.766 (0.657, 0.894) | 0.001^**^ | 0.732 (0.627, 0.856) | <0.001^***^ |
| Q4 | 0.758 (0.652, 0.882) | <0.001^***^ | 0.705 (0.601, 0.827) | <0.001^***^ | 0.690 (0.583, 0.817) | <0.001^***^ |
| Q5 | 0.960 (0.806, 1.143) | 0.643 | 0.893 (0.746, 1.069) | 0.216 | 0.838 (0.688, 1.022) | 0.081 |
| *P* for trend |  | 0.260 |  | 0.078 |  | 0.029^*^ |

“^*^”, *P*<0.05; “^**^”, *P*<0.01; “^***^”, *P*<0.001.
