# Supplementary material for: Adherence to the planetary health diet index and metabolic dysfunction-associated steatotic liver disease: a cross-sectional study
Source: Front Nutr. 2025 Feb 20;12:1534604. doi: 10.3389/fnut.2025.1534604 (PMC11882404; doi:10.3389/fnut.2025.1534604)
Supplement: Supplementary file 4 [file Table_4.docx]

| Supplementary Table S4 The threshold effects of PHDI components on MASLD using a two-stage phased regression model | | |
| --- | --- | --- |
| Models | Adjusted OR (95%CI) | *P* value |
| Non-starchy vegetables | | |
| Model I | | |
| logistic regression (the standard linear model) | 0.930(0.909-0.950) | <0.001^***^ |
| Model II | | |
| Inflection point | 4.883 |  |
| <4.883 | 0.923(0.897-0.950) | <0.001^***^ |
| >4.883 | 0.955(0.884-1.030) | 0.240 |
| P for likelihood ratio test |  | 0.464 |
| Whole fruits (exclude fruit juice) | | |
| Model I |  |  |
| logistic regression (the standard linear model) | 0.976(0.965-0.987) | <0.001^***^ |
| Model II |  |  |
| Inflection point | 7.575 |  |
| <7.575 | 0.960(0.945-0.976) | <0.001^***^ |
| >7.575 | 1.057(0.993-1.126) | 0.082 |
| P for likelihood ratio test |  | 0.011^*^ |
| Red and processed meat | | |
| Model I |  |  |
| logistic regression (the standard linear model) | 0.983(0.974-0.992) | <0.001^***^ |
| Model II |  |  |
| Inflection point | 1.221 |  |
| <1.221 | 0.860(0.751-0.984) | 0.028^*^ |
| >1.221 | 1.003(0.981-1.026) | 0.785 |
| P for likelihood ratio test |  | 0.050 |
| Soy products |  |  |
| Model I |  |  |
| logistic regression (the standard linear model) | 0.932(0.900-0.964) | <0.001^***^ |
| Model II |  |  |
| Inflection point | 0.250 |  |
| <0.250 | 1.792(0.956-3.343) | 0.068 |
| >0.250 | 0.892(0.846-0.941) | <0.001^***^ |
| P for likelihood ratio test |  | 0.041^*^ |
| Unsaturated fatty acids |  |  |
| Model I |  |  |
| logistic regression (the standard linear model) | 1.085(1.064-1.106) | <0.001^***^ |
| Model II |  |  |
| Inflection point | 9.541 |  |
| <9.553 | 1.054(1.026-1.083) | <0.001^***^ |
| >9.553 | 1.474(1.200-1.810) | <0.001^***^ |
| P for likelihood ratio test |  | 0.003^**^ |

| Supplementary Table 3 continued | | |
| --- | --- | --- |
| Models | Adjusted OR (95%CI) | *P* value |
| Saturated fatty acids | | |
| Model I | | |
| logistic regression (the standard linear model) | 0.884(0.862-0.906) | <0.001^***^ |
| Model II | | |
| Inflection point | 0.005 |  |
| <0.005 | 0.000(0.000-0.000) | 0.003^**^ |
| >0.005 | 0.917(0.886-0.950) | <0.001^***^ |
| P for likelihood ratio test |  | 0.003^**^ |
| Added sugar | | |
| Model I |  |  |
| logistic regression (the standard linear model) | 0.792(0.737-0.851) | <0.001^***^ |
| Model II |  |  |
| Inflection point | 9.561 |  |
| <9.561 | 0.983(0.881-1.098) | 0.763 |
| >9.561 | 0.360(0.264-0.492) | <0.001^***^ |
| P for likelihood ratio test |  | <0.001^***^ |

“*”: P<0.05; “**”: P<0.01;“***”: P<0.001.
