# Supplementary material for: Adherence to the planetary health diet index and metabolic dysfunction-associated steatotic liver disease: a cross-sectional study
Source: Front Nutr. 2025 Feb 20;12:1534604. doi: 10.3389/fnut.2025.1534604 (PMC11882404; doi:10.3389/fnut.2025.1534604)
Supplement: Supplementary file 5 [file Table_5.docx]

| Supplementary Table S5 The relationship between PHDI and MASLD using NHANES 2011-2018 | | | | | | |
| --- | --- | --- | --- | --- | --- | --- |
| Variable | Model 1 | | Model 2 | | Model 3 | |
|  | OR (95% CI) | *P* value | OR (95% CI) | *P* value | OR (95% CI) | *P* value |
| PHDI | 0.985 (0.981, 0.990) | <0.001^***^ | 0.983 (0.978, 0.987) | <0.001^***^ | 0.985 (0.980, 0.990) | <0.001^***^ |
| PHDI (Quintile) | | | | | | |
| Q1 | Ref |  | Ref |  | Ref |  |
| Q2 | 1.002 (0.814, 1.235) | 0.981 | 0.946 (0.758, 1.181) | 0.621 | 0.929 (0.728, 1.185) | 0.542 |
| Q3 | 0.959 (0.771, 1.193) | 0.701 | 0.892 (0.711, 1.120) | 0.318 | 0.812 (0.628, 1.051) | 0.110 |
| Q4 | 0.862 (0.685, 1.084) | 0.200 | 0.786 (0.625, 0.990) | 0.041^*^ | 0.776 (0.620, 0.970) | 0.027^**^ |
| Q5 | 0.584 (0.477, 0.714) | <0.001^***^ | 0.520 (0.419, 0.644) | <0.001^***^ | 0.565 (0.436, 0.731) | <0.001^***^ |
| *P* for trend | | <0.001^***^ |  | <0.001^***^ |  | <0.001^***^ |

“^*^”, *P*<0.05; “^**^”, *P*<0.01; “^***^”, *P*<0.001.
