# Supplementary material for: Adherence to the planetary health diet index and metabolic dysfunction-associated steatotic liver disease: a cross-sectional study
Source: Front Nutr. 2025 Feb 20;12:1534604. doi: 10.3389/fnut.2025.1534604 (PMC11882404; doi:10.3389/fnut.2025.1534604)
Supplement: Supplementary file 6 [file Table_6.docx]

| Supplementary Table S6 The association between PHDI and MASLD using unweighted data | | | | | | |
| --- | --- | --- | --- | --- | --- | --- |
| Variable | Model 1 | | Model 2 | | Model 3 | |
|  | OR (95% CI) | *P* value | OR (95% CI) | *P* value | OR (95% CI) | *P* value |
| PHDI | 0.990 (0.987, 0.992) | <0.001^***^ | 0.986 (0.984, 0.989) | <0.001^***^ | 0.988 (0.985, 0.990) | <0.001^***^ |
| PHDI (Quintile) | | | | | | |
| Q1 | Ref |  | Ref |  | Ref |  |
| Q2 | 1.026 (0.929, 1.134) | 0.610 | 0.984 (0.889, 1.090) | 0.760 | 0.998 (0.894, 1.113) | 0.965 |
| Q3 | 0.975 (0.882, 1.078) | 0.627 | 0.893 (0.805, 0.990) | 0.031^*^ | 0.883 (0.790, 0.986) | 0.027^*^ |
| Q4 | 0.922 (0.834, 1.019) | 0.112 | 0.824 (0.743, 0.915) | 0.001^**^ | 0.834 (0.745, 0.932) | 0.001^**^ |
| Q5 | 0.664 (0.599, 0.736) | <0.001^***^ | 0.584 (0.524, 0.650) | <0.001^***^ | 0.617 (0.549, 0.694) | <0.001^***^ |
| *P* for trend | | <0.001^***^ |  | <0.001^***^ |  | <0.001^***^ |

“^*^”, *P*<0.05; “^**^”, *P*<0.01; “^***^”, *P*<0.001.
