# Supplementary material for: Adherence to the planetary health diet index and metabolic dysfunction-associated steatotic liver disease: a cross-sectional study
Source: Front Nutr. 2025 Feb 20;12:1534604. doi: 10.3389/fnut.2025.1534604 (PMC11882404; doi:10.3389/fnut.2025.1534604)
Supplement: Supplementary file 7 [file Table_7.docx]

| Supplementary Table S7 The association between PHDI and MASLD using unimputed data | | | | | | |
| --- | --- | --- | --- | --- | --- | --- |
| Variable | Model 1 | | Model 2 | | Model 3 | |
|  | OR (95% CI) | *P* value | OR (95% CI) | *P* value | OR (95% CI) | *P* value |
| PHDI | 0.985 (0.981, 0.989) | <0.001^***^ | 0.983 (0.980, 0.987) | <0.001^***^ | 0.985 (0.981, 0.989) | <0.001^***^ |
| PHDI (Quintile) | | | | | | |
| Q1 | Ref |  | Ref |  | Ref |  |
| Q2 | 1.027 (0.854, 1.235) | 0.775 | 0.992 (0.822, 1.198) | 0.935 | 0.988 (0.805, 1.212) | 0.904 |
| Q3 | 0.975 (0.817, 1.163) | 0.777 | 0.927 (0.777, 1.108) | 0.401 | 0.857 (0.705, 1.041) | 0.118 |
| Q4 | 0.805 (0.659, 0.983) | 0.034^*^ | 0.755 (0.617, 0.923) | 0.007^**^ | 0.720 (0.592, 0.876) | 0.001^**^ |
| Q5 | 0.573 (0.479, 0.685) | <0.001^***^ | 0.538 (0.449, 0.644) | <0.001^***^ | 0.564 (0.457, 0.697) | <0.001^***^ |
| *P* for trend | | <0.001^***^ |  | <0.001^***^ |  | <0.001^***^ |

“^*^”, *P*<0.05; “^**^”, *P*<0.01; “^***^”, *P*<0.001.
