# Supplementary material for: Adherence to the planetary health diet index and metabolic dysfunction-associated steatotic liver disease: a cross-sectional study
Source: Front Nutr. 2025 Feb 20;12:1534604. doi: 10.3389/fnut.2025.1534604 (PMC11882404; doi:10.3389/fnut.2025.1534604)
Supplement: Supplementary file 8 [file Table_8.docx]

| Supplementary Table S8 The relationship between PHDI and MASLD using 1 day diet data | | | | | | |
| --- | --- | --- | --- | --- | --- | --- |
| Variable | Model 1 | | Model 2 | | Model 3 | |
|  | OR (95% CI) | *P* value | OR (95% CI) | *P* value | OR (95% CI) | *P* value |
| PHDI | 0.986 (0.982, 0.990) | <0.001^***^ | 0.984 (0.980, 0.988) | <0.001^***^ | 0.984 (0.980, 0.989) | <0.001^***^ |
| PHDI (Quintile) | | | | | | |
| Q1 | Ref |  | Ref |  | Ref |  |
| Q2 | 0.940 (0.791, 1.117) | 0.476 | 0.935 (0.785, 1.115) | 0.452 | 1.016 (0.814, 1.268) | 0.885 |
| Q3 | 0.961 (0.825, 1.118) | 0.601 | 0.933 (0.803, 1.084) | 0.362 | 1.011 (0.833, 1.228) | 0.907 |
| Q4 | 0.791 (0.686, 0.913) | 0.002^**^ | 0.749 (0.650, 0.864) | <0.001^***^ | 0.738 (0.608, 0.896) | 0.003^**^ |
| Q5 | 0.562 (0.473, 0.667) | <0.001^***^ | 0.520 (0.437, 0.619) | <0.001^***^ | 0.540 (0.439, 0.663) | <0.001^***^ |
| *P* for trend | | <0.001^***^ |  | <0.001^***^ |  | <0.001^***^ |

“^*^”, *P*<0.05; “^**^”, *P*<0.01; “^***^”, *P*<0.001.
