# Supplementary material for: Adherence to the planetary health diet index and metabolic dysfunction-associated steatotic liver disease: a cross-sectional study
Source: Front Nutr. 2025 Feb 20;12:1534604. doi: 10.3389/fnut.2025.1534604 (PMC11882404; doi:10.3389/fnut.2025.1534604)
Supplement: Supplementary file 11 [file Supplement_file_1.docx]

1. Racial groups were categorized as Non-Hispanic White, Non-Hispanic Black, Mexican American, and Other race.
2. The PIR was divided into three categories: low (≤1.3), medium (1.3-3.5), and high (>3.5).
3. Educational attainment was grouped into less than high school, high school or equivalent, and some college or more.
4. BMI was categorized as normal weight (<25.0 kg/m²), overweight (25.0-30.0 kg/m²), and obese (>30.0 kg/m²).
5. Smoking status was assessed using NHANES data, identifying individuals who had smoked at least 100 cigarettes in their lifetime as smokers, with further classification into now, former, or never smokers.
6. Diabetes was defined based on the use of insulin or oral hypoglycemic drugs, self-reported diabetes, or fasting glucose levels of ≥7.0 mmol/L or HbA1c ≥6.5%.
7. Hypertension was diagnosed if systolic blood pressure was ≥140 mmHg or diastolic ≥90 mmHg, or if there was a history of hypertension or use of antihypertensive drugs.
8. Cardiovascular outcomes included a history of heart failure, coronary artery disease, angina, or myocardial infarction.
